# Biosensor and chemo-enzymatic one-pot cascade applications to detect and transform PET-derived terephthalic acid in living cells

**DOI:** 10.1016/j.isci.2022.104326

**Published:** 2022-04-29

**Authors:** Thomas Bayer, Lara Pfaff, Yannick Branson, Aileen Becker, Shuke Wu, Uwe T. Bornscheuer, Ren Wei

**Affiliations:** 1 Institute of Biochemistry, Department of Biotechnology & Enzyme Catalysis, University of Greifswald, Felix-Hausdorff-Straße 4, 17487 Greifswald, Germany; 2 Institute of Molecular Biotechnology, TU Graz, Petersgasse 14, 8010 Graz, Austria; 3 College of Life Science & Technology, Huazhong Agricultural University, Shizishan Street 1, Wuhan 430070, China

**Keywords:** Sensor, Polymer chemistry, Enzyme engineering, Bioelectronics

## Abstract

Plastic waste imposes a serious problem to the environment and society. Hence, strategies for a circular plastic economy are demanded. One strategy is the engineering of polyester hydrolases toward higher activity for the biotechnological recycling of polyethylene terephthalate (PET). To provide tools for the rapid characterization of PET hydrolases and the detection of degradation products like terephthalic acid (TPA), we coupled a carboxylic acid reductase (CAR) and the luciferase LuxAB. CAR converted TPA into the corresponding aldehydes in *Escherichia coli*, which yielded bioluminescence that not only semiquantitatively reflected amounts of TPA in hydrolysis samples but is suitable as a high-throughput screening assay to assess PET hydrolase activity. Furthermore, the CAR-catalyzed synthesis of terephthalaldehyde was combined with a reductive amination cascade in a one-pot setup yielding the corresponding diamine, suggesting a new strategy for the transformation of TPA as a product obtained from PET biodegradation.

## Highlights

• First bioreduction of terephthalic acid (TPA) by a carboxylic acid reductase *in vivo*
• Real-time, high-throughput detection of TPA-derived aldehydes by luciferase LuxAB
• Bioluminescence reflects TPA amounts, assessing (engineered) PET hydrolase activity
• Transformation of TPA into the diamine through chemo-enzymatic one-pot cascade

## Graphical abstract

**Figure:**
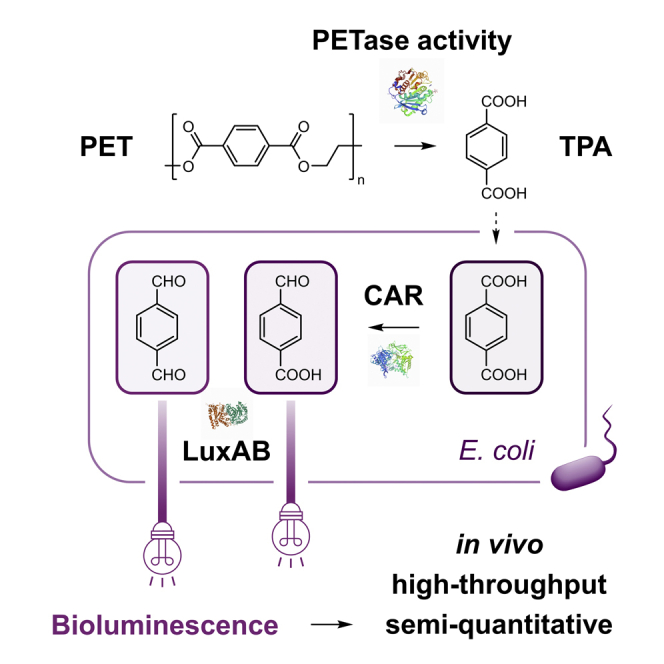

## Introduction

The global production of plastics is rapidly increasing. More than 8% of the global petrochemical production – 4% as source for materials and 4% to cover energy demands – were consumed by plastic manufacturing industries (Hopewell et al., 2009). However, only a fraction of discarded plastic is recycled (Geyer et al., 2017). Consequently, efficient disposal and sustainable recycling strategies for plastic waste are urgently needed to reduce the risk of pollution imposed on ecosystems and human health (Eriksen et al., 2014; Rahimi and García, 2017; Vollmer et al., 2020; Wright and Kelly, 2017). Furthermore, to decrease both carbon dioxide (CO_2_) emissions and the dependence on fossil fuel-based resources, a circular plastic economy is regarded as the central – and vital – approach (Raoul et al., 2021; Sarah and Gloria, 2021; Simon et al., 2021; Wei et al., 2020).

Particularly, the biocatalysis-based recycling of polyethylene terephthalate (PET), which is extensively used to manufacture food packaging and beverage containers, has become a vivid field of research with the discovery of microbial PET-degrading enzymatic activities (Kawai et al., 2019, 2020; Tournier et al., 2020; Wei et al., 2020, 2022; Yoshida et al., 2016). So far, PET hydrolases from actinomycetes including different *Thermobifida* strains (Herrero Acero et al., 2011; Müller et al., 2005; Wei et al., 2014), from the bacterium *Ideonella sakaiensis* (Yoshida et al., 2016), and a commercial cutinase from the fungi *Thermomyces insolens*, formerly known as *Humicola insolens*, have been employed (Ronkvist et al., 2009). Recently, a variant of the compost metagenome-derived and highly thermostable leaf-branch compost cutinase (LCC) (Sulaiman et al., 2012) was engineered toward increased PET-hydrolyzing activity, which pushed the enzymatic depolymerization of PET from laboratory scales to industrially relevant metrics by degrading amorphized (i.e., pretreated) postconsumer PET bottles in only 10 h reaction time (Tournier et al., 2020). This and the fact that LCC as well as other PET hydrolases were found in public metagenome databases will certainly advance biotechnological plastic degradation and recycling in the near future (Bornscheuer, 2016; Danso et al., 2018; Wei et al., 2020, 2022).

Despite the many achievements in the last two decades, the activity of PET hydrolases is still assessed by simply measuring the weight loss of the residual bulk PET polymer after depolymerization (Wei et al., 2019a, 2019b; Yoshida et al., 2016) or the chromatographic analysis and quantification of degradation intermediates and/or products such as terephthalic acid (TPA) and its monoesters and diesters (Eberl et al., 2009; Herrero Acero et al., 2011; Palm et al., 2019). Recently, an isothermal titration calorimetry-based method has been established for directly assessing the enthalpy of ester hydrolysis, thus enabling a real-time monitoring of the enzymatic PET hydrolysis (Vogel et al., 2021). All these strategies suffer from laborious sample preparation and the only low to moderate sample throughput, impeding the characterization of novel biocatalysts – not only limited to polyester hydrolases – and the screening of large protein libraries (Markel et al., 2020; Wei et al., 2020, 2022; Yi et al., 2021). This obstacle was addressed by a Fenton chemistry-mediated fluorometric detection assay for TPA in a 96-well microtiter plate format, suitable for high-throughput (HT) screening applications (Pfaff et al., 2021; Wei et al., 2012). The assay is based on the formation of hydroxyl radicals mediated by an Fe(II)-ethylenediaminetetraacetic acid complex in the presence of molecular oxygen (O_2_) (Saran and Summer, 1999; Wei et al., 2012; Welch et al., 2002); hydroxyl radicals and TPA then react to the fluorescent 2-hydroxyterephthalate (λ_excitation_ = 315 nm, λ_emission_ = 421 nm).

Complementary, genetically encoded biosensor systems have been used for the detection of small molecules and included transcription factors (TFs), riboswitches, or enzyme-coupled sensor devices (Bayer et al., 2021; Dietrich et al., 2010; Lehtinen et al., 2017; Liu et al., 2015, 2017; Yi et al., 2021). To date, only two biosensors have been reported to detect TPA *in vivo*. The first was assembled by Pardo et al. and comprised the TF TphR and its regulatory nucleotide sequences from *Comamonas testosteroni* and the superfolder green fluorescent protein (sfGFP) (Pardo et al., 2020). TphR is a transcriptional activator, which – upon binding of TPA – acts as the inducer of a gene cluster responsible for the conversion of TPA to protocatechuate in *Comamonas* strains (Kasai et al., 2010). Their TF-based biosensor system facilitated the screening of TPA transporter variants, in other words, the improved uptake of TPA from the environment in *Acinetobacter baylyi* ADP1 through fluorescence-activated cell sorting (Pardo et al., 2020). The second example featured sfGFP as the fluorescence reporter and the promiscuous TF XylS from *Pseudomonas putida*, which was engineered by Li and coworkers to bind TPA additionally to reported benzoic acid derivatives (Li et al., 2022). With the efficient detection of TPA in living cells, these sensing devices have yet to be tested for the directed evolution of PET hydrolases by the HT-assisted detection of TPA as PET degradation product.

**Figure 1:**
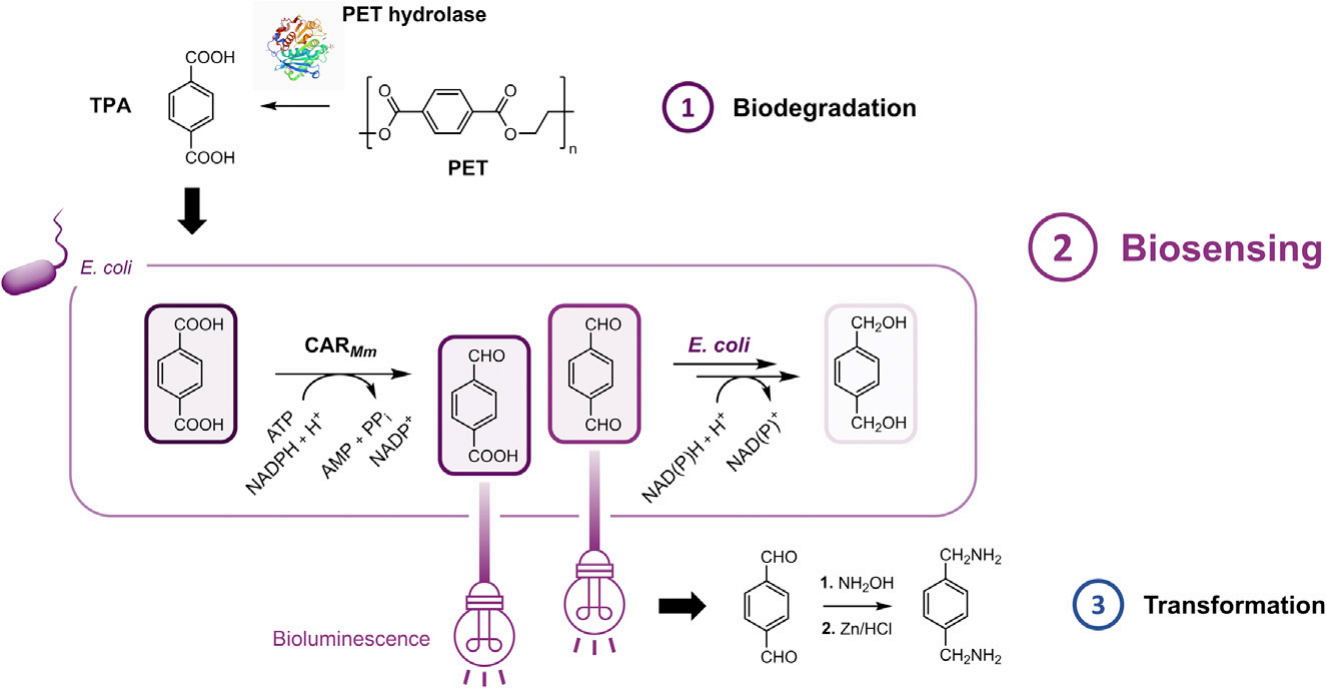
Enzyme-coupled biosensor for the detection of TPA in *E. coli* (1) The biocatalytic degradation of PET by hydrolases releases monomeric molecules including TPA and ethylene glycol (not shown). The PET hydrolase structure in the scheme was adapted from PDB: 6THT (Tournier et al., 2020). (2) TPA can be reduced to the corresponding dialdehydes and monoaldehydes by CAR_*Mm*_ (accessory PPT_*Ni*_ not shown). These aldehydes are sensed by LuxAB, thereby emitting bioluminescence. Endogenous enzymes further reduce aldehydes to the corresponding primary alcohols. (3) The reactive TAL can be captured as aldoxime (not shown) and further converted to the diamine by reductive amination and basic work-up in a one-pot cascade, interconverting polymer precursors as future upcycling option after further optimization.

Most recently, the luciferase LuxAB from *Photorhabdus luminescens* (*P. luminescens*) was introduced for the detection of structurally diverse aldehydes in *Escherichia coli* (*E. coli*) (Bayer et al., 2021). In the present work, the carboxylic acid reductase from *Mycobacterium marinum* (CAR_*Mm*_) was shown to transform TPA into the corresponding aldehydes 4-carboxybenzaldehyde (4-CBAL) and terephthalaldehyde (TAL) *in vivo* (Figure 1). The coupling of the enzymatic reduction to the LuxAB biosensor device yielded bioluminescence that semiquantitatively reflected increasing amounts of TPA in PET hydrolysis samples obtained through various hydrolases. The system not only provides a biosensor-based HT assay for TPA but the first biocatalytic route toward highly reactive TPA-derived aldehydes such as TAL, avoiding hazardous chemical procedures (Barnicki, 2017; Snell and Weissberger, 1940). Following the transformation of TAL in the same reaction vessel, the corresponding diamine was yielded and will allow for potential industrial applications (Brindell et al., 1976; Rohan et al., 2015; Suematsu et al., 1983; Wang et al., 2021).

## Results

### Optimization of whole-cell biotransformations and evaluation of HT assay conditions

In a previous study, the monooxygenase LuxAB from *P. luminescens* was expressed in *E. coli* K-12 MG1655 RARE (Kunjapur et al., 2014), herein referred to as *E. coli* RARE, and provided a reliable detection tool for aldehydes in living cells in a 96-well microplate format (Bayer et al., 2021), importantly, beyond the previously reported long-chain aliphatic aldehydes (Colepicolo et al., 1989). Furthermore, LuxAB was suitable to sense aldehydes, including aromatic products such as benzaldehyde, cuminaldehyde, and 2-phenylacetaldehyde that were enzymatically produced from carboxylic acid substrates by the co-expression of CAR_*Mm*_ in the same cell (Bayer et al., 2021). Prompted by the structural relatedness of these aromatic aldehydes to TPA-derived aldehydes, the capabilities of (1) CAR_*Mm*_ – to reduce one or both carboxylic acid functionalities of TPA to the aldehyde – and (2) LuxAB – to accept aldehyde products formed *in situ*, thereby yielding bioluminescence – were investigated.

**Figure 2:**
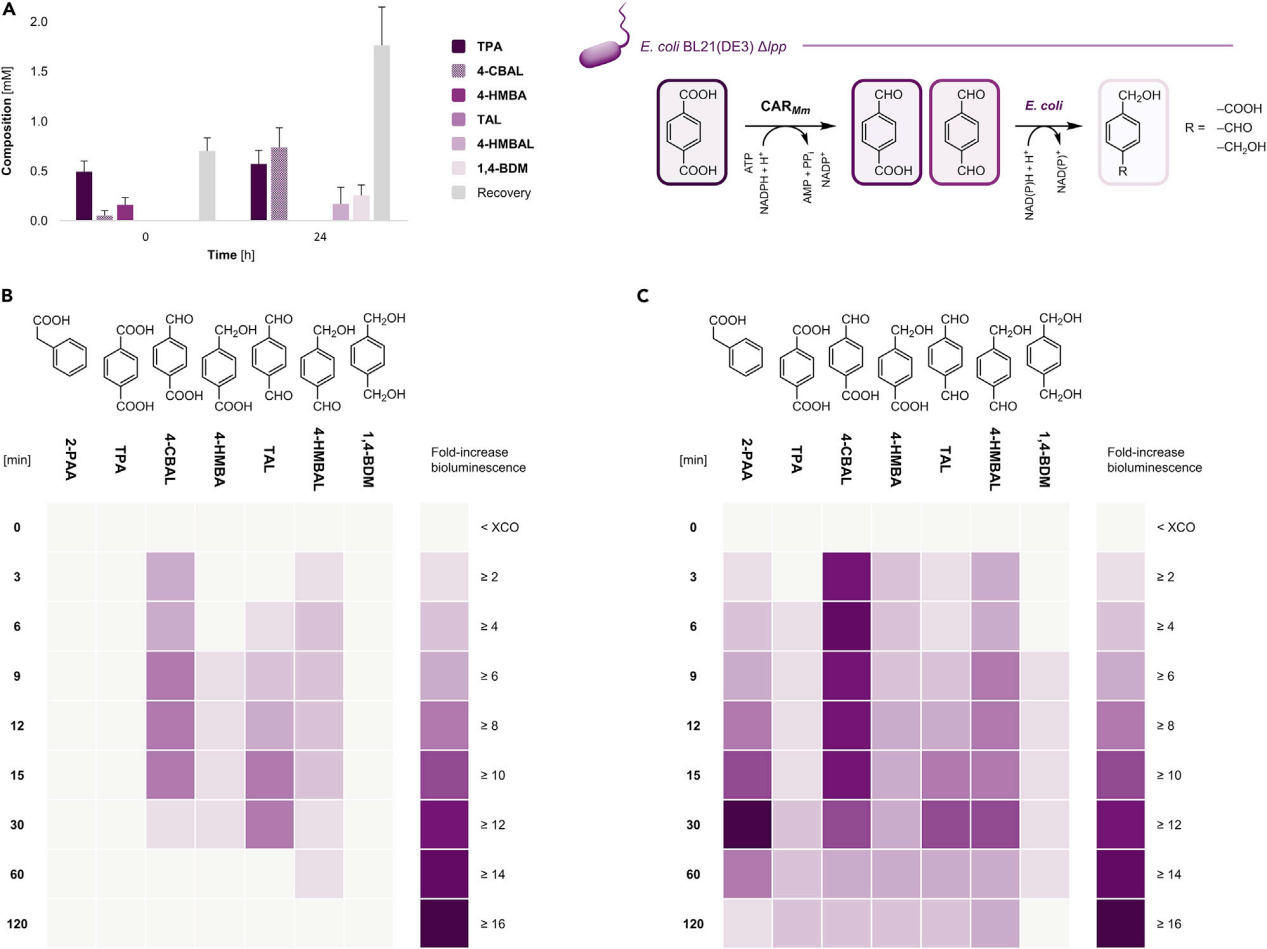
Enzyme-coupled biosensor assembly in *E. coli* BL21(DE3) Δ*lpp* (A) CAR_*Mm*_ reduces TPA to 4-CBAL and TAL, which are further reduced to 4-HMBA, 4-HMBAL, and 1,4-BDM by endogenous enzymes *in vivo*; PPT_*Ni*_ for posttranslational modification of CAR_*Mm*_ is omitted for clarity. Experiments were performed in RCs of *E. coli* BL21(DE3) Δ*lpp* (OD_600_ ≈ 10.0) co-expressing enzymes from pACYCDuet-1/*car*_*Mm*_*:ppt*_*Ni*_ (Bayer et al., 2021) in the presence of 2 mM TPA and 5% (*ν/ν*) DMSO as organic cosolvent. Sampling: 0 h (after the addition of TPA and mixing) and 24 h. Recoveries were reduced because of low solubility of TPA in resting cell medium (RCM) and the volatility of reaction compounds. GC yields are presented as mean values + standard deviation (SD) [mM] of biological replicates (n = 3); see also Figure S1. (B) Direct detection of aldehydes (1 mM) by increasing bioluminescence over time in RCs of *E. coli* BL21(DE3) Δ*lpp* expressing LuxAB from pLuxAB. (C) *In situ* production of aldehydes from carboxylates (1 mM) in RCs of *E. coli* BL21(DE3) Δ*lpp* co-expressing LuxAB and CAR_*Mm*_/PPT_*Ni*_; 2-phenyl acetic acid (2-PAA) was used as control. Experiments were performed in the presence of 1% (*ν/ν*) DMSO under HT assay conditions as described previously (Bayer et al., 2021); data presented as mean fold-increase bioluminescence obtained from biological replicates (n = 3). For results employing *E. coli* RARE, see Figure S3.

Therefore, chemically competent *E. coli* BL21(DE3) cells were transformed with pACYCDuet-1/*car*_*Mm*_*:ppt*_*Ni*_ to co-express CAR_*Mm*_ and a phosphopantetheinyl transferase from *Nocardia iowensis* (PPT_*Ni*_) (Bayer et al., 2021). The PPT is required to posttranslationally modify apoCARs to yield the functional holo-CAR enzymes (Akhtar et al., 2013; Finnigan et al., 2017; Horvat and Winkler, 2020). Whereas TPA was not converted in resting cells (RCs) of untransformed *E. coli*, the detection of 4-(hydroxymethyl) benzaldehyde (4-HMBAL) and 1,4-benzenedimethanol (1,4-BDM; 32.7 ± 3.5% combined yields) by gas chromatography equipped with a flame ionization detector (GC/FID) indicated both the activity of CAR_*Mm*_ toward TPA and the further reduction of aldehydes by endogenous host enzymes (Figure 1 and Table 1) (Bayer et al., 2017; Kunjapur et al., 2014; Kunjapur and Prather, 2015). However, biotransformation mixtures contained up to 75% unreacted TPA besides the over-reduced products after 24 h (Figure S1A). Although a similar conversion of TPA was achieved with RCs of *E. coli* RARE (Figure S1B), the utilization of *E. coli* BL21(DE3) Δ*lpp* enhanced the bioreduction of TPA significantly (Figure 2A). RCs of the engineered strain harboring pACYCDuet-1/*car*_*Mm*_*:ppt*_*Ni*_ were prepared and biotransformations were carried out as outlined below. The resulting suspension contained a mixture of TPA (31.1 ± 5.9%), 4-CBAL (36.8 ± 9.9%), 4-HMBAL (6.5 ± 2.2%), and 1,4-BDM (13.7 ± 5.7%) according to GC/FID (Figure 2A); the highly reactive TAL could only be detected in traces. The nonessential *lpp* gene encodes one of the most abundant cellular proteins in terms of copy number (Li et al., 2014) and controls the (mechanical) properties of the inner and outer membrane (Asmar et al., 2017; Mathelié-Guinlet et al., 2020). Not only was its deletion suggested to affect the permeability of the cellular envelope for small molecules (Ni et al., 2007); it increased expression levels of CAR_*Mm*_ according to sodium dodecyl sulfate-polyacrylamide gel electrophoresis (SDS-PAGE) analysis (Figure S2). This may be explained by the reallocation of cellular resources (Li et al., 2014) and might provide a general approach to improve heterologous protein production.

**Table 1:** List of compounds

Compound (Abbreviation)	Retention time [min]	RRF
Terephthalic acid (TPA)	6.90–7.00	0.243
4-Carboxybenzaldehyde (4-CBAL)	3.70–3.80	0.297
4-(Hydroxymethyl) benzoic acid (4-HMBA)	4.14	0.216
Terephthalaldehyde (TAL)	4.23	0.932
4-(Hydroxymethyl) benzaldehyde (4-HMBAL)	5.22	1.132
1,4-Benzenedimethanol (1,4-BDM)	5.51	1.168
1,4-bis-(Aminomethyl) benzene (1,4-bis-AMB)	5.27	0.830
Benzylamine (BAM)	2.76	0.783
Methyl benzoate (IS)	3.28	–

The retention times for benzoic acid and 2-phenylacetic acid and their corresponding aldehydes and primary alcohols as well as GC/FID-based quantification were reported previously (Bayer et al., 2021). Relative response factors (RFFs) were used as mean values of independently prepared standard solutions (n ≥ 3) analyzed by GC/FID.

Subsequently, RCs of *E. coli* BL21(DE3) Δ*lpp* as well as *E. coli* RARE were prepared either expressing only LuxAB or the luciferase together with CAR_*Mm*_/PPT_*Ni*_. *E. coli* RARE exhibits reduced aromatic aldehyde-reducing activity (Kunjapur et al., 2014) and has been employed by various groups to increase the persistence of aldehydes for both their production *in vivo* (Bayer et al., 2017; Horvat and Winkler, 2020; Kunjapur et al., 2016) and their efficient detection (Bayer et al., 2021; Ressmann et al., 2019).

Satisfyingly, the previously established HT assay conditions yielded bioluminescence in the presence of TPA-derived aldehydes in both *E. coli* strains expressing LuxAB (Bayer et al., 2021). At 1 mM final concentration, the highest fold-increase in bioluminescence was observed in the presence of 4-CBAL and TAL, both elevating bioluminescence about 8-fold above background in RCs of *E. coli* BL21(DE3) Δ*lpp* after 15 min, followed by 4-HMBAL (4-fold) (Figure 2B). As expected, TPA did not increase bioluminescence in RCs only expressing the biosensor, but signals increased more than 4-fold when co-expressing LuxAB and CAR_*Mm*_/PPT_*Ni*_ in the same cell (Figure 2C). Similar results were obtained with RCs of *E. coli* RARE upon the addition of TPA (Figure S3; 1 mM final concentration); to extenuate the cytotoxic effects of initially high aldehyde levels and in accordance with previous findings, 4-CBAL, TAL, and 4-HMBAL could be efficiently detected at 0.1 mM final concentration in *E. coli* RARE (Figure S3) (Bayer et al., 2021). The established reduction of 2-phenylacetic acid to 2-phenylacetaldehyde by CAR_*Mm*_ was included as positive control for the HT assay because the latter is accepted by LuxAB (Figure 2B–2C). DMSO slightly increased background luminescence over time, which had also been shown for other cosolvents like ethanol and acetonitrile (Bayer et al., 2021). Supporting the results of the HT assay (Figure 2C), the activity of the CAR enzyme toward 4-CBAL and 4-HMBA could be confirmed by GC/FID analysis of extracts from biotransformations employing CAR_*Mm*_/PPT_*Ni*_ (Figures S1C–S1D).

Motivated by the functional CAR/luciferase biosensor couple for the detection of TPA, the assay was tested with hydrolysate samples obtained after the enzymatic degradation of PET.

### Assaying TPA in PET hydrolysis samples under HT conditions

For the preparation of PET hydrolysates, the codon-optimized genes of LCC, the engineered variant LCC-ICCG (Tournier et al., 2020), and the polyester hydrolase-1 (PES-H1) (Zimmermann et al., 2019) were expressed from pET26b vectors in *E. coli* BL21(DE3) cultivated in auto-induction medium (AIM) supplemented with kanamycin and finally purified as described in this study.

**Figure 3:**
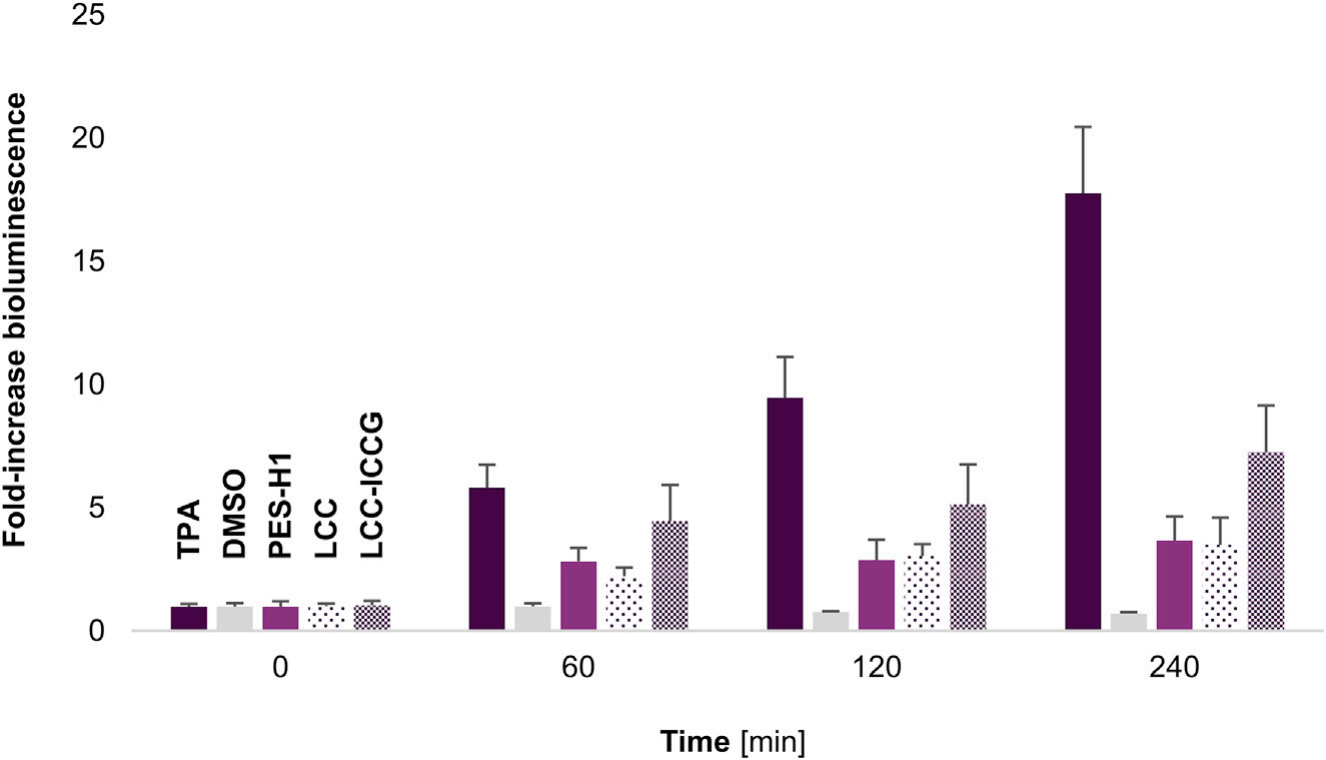
PET hydrolysis samples analyzed under HT conditions in *E. coli* RARE The enzyme-coupled biosensor system yielded bioluminescence in the presence of 1 mM TPA (positive control) and hydrolysates obtained by the enzymatic degradation of Gf-PET films by PES-H1, LCC, and LCC-ICCG; the bioluminescence did not increase in the presence of 1% (*ν/ν*) DMSO over monitoring time. Experiments were performed in RCs of *E. coli* RARE under HT assay conditions as described previously (Bayer et al., 2021); data presented as mean values of the fold-increase in bioluminescence + SD of biological replicates (n ≥ 3). For results employing *E. coli* BL21(DE3) Δ*lpp* RCs, see Figure S4.

The enzymatic degradation of amorphous PET film (Gf-PET, purchased from Goodfellow Ltd.) by LCC, LCC-ICCG, and PES-H1 was adapted from Tournier et al. as outlined below (Tournier et al., 2020). Hydrolysates were processed as described in this study and analyzed by the CAR_*Mm*_/LuxAB biosensor system under HT conditions (Figure 3) as well as calibrated high-performance liquid chromatography (HPLC; Table S2).

In the presence of 1 mM TPA, the bioluminescence increased about 4-fold and 5-fold in RCs of *E. coli* BL21(DE3) Δ*lpp* and *E. coli* RARE, respectively, after 1 h. While the fold-increase in bioluminescence plateaued in *E. coli* BL21(DE3) Δ*lpp* for 4 h (Figure S4), it increased more than 17-fold in *E. coli* RARE cells during the same reaction time (Figure 3). This difference can be explained by the distinct metabolic backgrounds of the two strains as highlighted earlier. The knockout of several alcohol dehydrogenases and aldo-keto reductases in *E. coli* RARE increases the persistence of (aromatic) aldehydes *in vivo*, including TPA-derived aldehydes (Kunjapur et al., 2014). In contrast, the activity of these endogenous enzymes in *E. coli* BL21(DE3) Δ*lpp* continuously reduces reactive aldehydes to the corresponding primary alcohols such as 1,4-BDM (Figure S1), which is not a substrate for LuxAB (Figure 2B).

Whereas bioluminescence signals were elevated >3-fold with PET hydrolysates obtained by the wild-type enzymes PES-H1 and LCC, bioluminescence increased >7-fold in LCC-ICCG samples after 4 h (Figure 3). This may be attributed to higher concentrations of potassium terephthalate salts in PET hydrolysates obtained by the LCC-ICCG variant compared to LCC, for example. Based on the fold-increases, TPA concentrations in the supernatants of the three hydrolysates were calculated and suggested 38.3 ± 3.8 mM, 39.3 ± 1.3 mM, and 95.5 ± 10.7 mM for PES-H1, LCC, and LCC-ICCG, respectively. Similar TPA yields in the same concentration range (56 mM, 47 mM, and 111 mM, respectively) were determined by HPLC (Table S2).

Given that the biosensor system is operating in living cells, the cytotoxicity of both carboxylates and the corresponding aldehydes (Bayer et al., 2017, 2021; Kunjapur and Prather, 2015), as well as the transient nature of bioluminescence signals (Fleiss and Sarkisyan, 2019) may interfere with the quantitative determination, allowing for marginal deviations from HPLC data. Nonetheless, the analysis of PET hydrolysates under HT conditions employing RCs of *E. coli* RARE yielded a reproducible fold-increase in bioluminescence based on the enzymatic transformation of TPA into the corresponding aldehydes and their detection by LuxAB, ultimately, reflecting TPA concentrations in PET hydrolysate samples semiquantitatively.

### Transformation of TAL by a chemo-enzymatic cascade in one pot

The chemical synthesis of (aromatic) aldehydes can be troublesome because of the high reactivity of the carbonyl group (Ferguson, 1946; Kunjapur and Prather, 2015). A promising alternative to specifically synthesize aldehydes are the well-established enzymatic reductions of carboxylates by CARs (Bayer et al., 2017, 2021; Butler and Kunjapur, 2020; Finnigan et al., 2017; Horvat and Winkler, 2020; Qu et al., 2018). CAR_*Mm*_ readily accepts TPA as indicated by the bioluminescence signals in the LuxAB-based HT assay (Figures 2C and S2B) and confirmed by the detection of 4-CBAL and TAL as intermediates and the corresponding over-reduced compounds 4-HMBAL and 1,4-BDM according to GC/FID. 4-HMBAL and 1,4-BDM are exclusively formed by the endogenous activities of host enzymes (Bayer et al., 2017; Kunjapur et al., 2014) (Figures 2A and S1). To the best of our knowledge, the CAR-catalyzed reduction of TPA is the first reported biocatalytic route forming TPA-derived aldehydes such as TAL, substituting hazardous chemical procedures (Snell and Weissberger, 1940). Depending on the purity and downstream application of plastic monomers from biocatalytic degradations, not all TPA is suitable for the resynthesis of virgin PET. Therefore, (bio)chemical transformation strategies for the re-use (*i.e.*, upcycling) of plastic precursors is of interest (Tiso et al., 2021). Recently, Sadler and Wallace synthesized vanillin from hydrolyzed waste PET by combining TPA-transforming enzymes from *Comamonas* sp. to yield intermediate catechol that was converted to the product by the activities of a CAR and an engineered catechol *O*-methyltransferase in *E. coli* RARE (Kunjapur and Prather, 2019; Sadler and Wallace, 2021).

**Figure 4:**
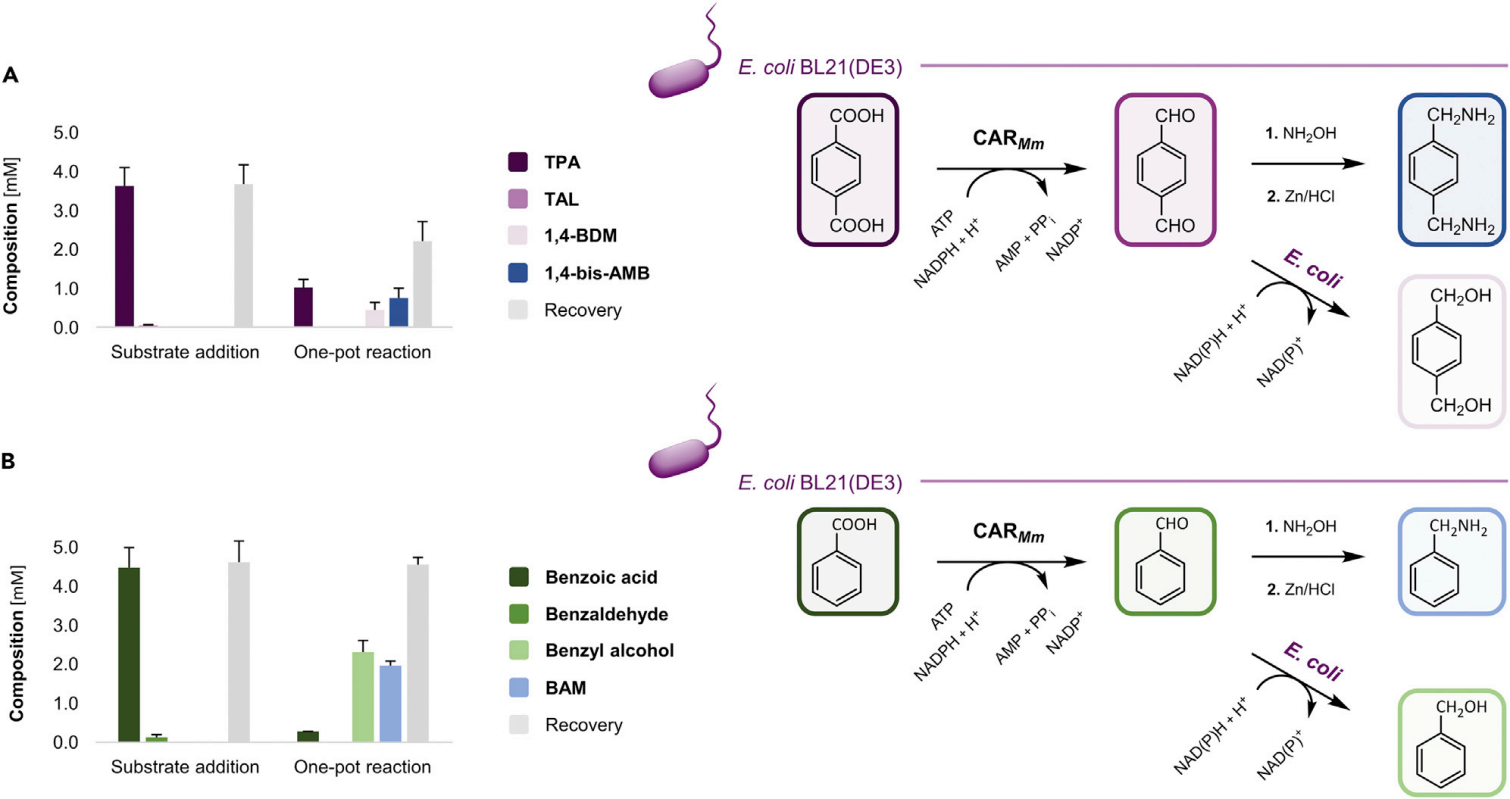
Chemo-enzymatic one-pot cascades Carboxylates are reduced by CAR_*Mm*_ in RCs of *E. coli* BL21(DE3) to the corresponding aldehydes; PPT_*Ni*_ is omitted for clarity. In the presence of NH_2_OH · HCl, the oximes are formed (not shown), which are reduced to the primary amines (shades of blue) after the addition of Zn/HCl to the same reaction vessel. (A) The TAL intermediate yields the desired 1,4-bis-AMB, besides 1,4-BDM as the major byproduct. Recoveries were reduced due to low solubility of TPA in RCM containing 5% (*ν/ν*) DMSO as organic co-solvent, the volatility of reaction compounds, and the formation of yet to be identified byproducts such as imines (Godoy-Alcántar et al., 2005; Simion et al., 2001). (B) Benzoic acid in the presence of 5% (*ν/ν*) ethanol was reduced to benzaldehyde, yielding the desired BAM after reductive amination and benzyl alcohol as the sole byproduct. Experiments were performed in RCs (OD_600_ ≈ 10.0) co-expressing enzymes from pACYCDuet-1/*car*_*Mm*_*:ppt*_*Ni*_ (Bayer et al., 2021). Sampling: (1) after the addition of NH_2_OH · HCl (2.2 and 1.1 equiv for TPA and benzoic acid, respectively) and carboxylic acid and mixing; (2) after performing the reductive amination in one-pot. GC yields are presented as mean values + SD [mM] of biological replicates (n = 3). Performance was similar with RCs of *E. coli* RARE producing 27.2 ± 6.6% BAM and 13.1 ± 8.0% 1,4-bis-AMB (n = 2).

In the following proof-of-concept example, benzaldehyde and TAL were produced from benzoic acid and TPA, respectively, by CAR_*Mm*_/PPT_*Ni*_ in *E. coli* BL21(DE3) or *E. coli* RARE RCs. The aldehydes were quenched in the presence of an excess of hydroxylamine hydrochloride (NH_2_OH · HCl) to form the corresponding aldoximes. Subsequently, reductive amination was performed in one pot by the addition of zinc powder and acidification (Ayedi et al., 2013). After extraction under basic conditions, the expected primary amines – benzylamine (BAM; 35.3 ± 0.7%) and 1,4-bis-(aminomethyl) benzene (1,4-bis-AMB; 15.0 ± 5.0%) – could be detected by GC/FID (Figure 4); benzyl alcohol and 1,4-BDM, respectively, were the major byproducts. Structurally related diamines find applications in synthesis of polyurethanes and polyamides, for example (Wang et al., 2021). In addition, although not further investigated in this study, the formation of imines might contribute to the low yield and the poor recovery of material in reactions starting from TPA (Godoy-Alcántar et al., 2005; Simion et al., 2001).

## Discussion

The expanding number of new PET hydrolases from natural resources including metagenomes as well as protein engineering endeavors calls for tools for their rapid characterization (Wei et al., 2022; Wiltschi et al., 2020). Furthermore, the functional assessment of these enzymes depends – with very few exceptions (Pfaff et al., 2021; Vogel et al., 2021) – on chromatographic methods characterized by only modest sample throughputs (Markel et al., 2020; Wei et al., 2020; Yi et al., 2021). To address this issue, this work coupled the activity of CAR_*Mm*_ to reduce TPA in different *E. coli* strains to the corresponding aldehydes (4-CBAL, TAL, and 4-HMBAL) with the genetically encoded biosensor LuxAB from *P. luminescens*. The latter emits detectable bioluminescence in the presence of TPA-derived aldehydes (Figures 1, 2 and S3). As TPA is a building block of PET, the CAR/LuxAB couple was employed to detect terephthalates in hydrolysate samples obtained from PET degradation, catalyzed by the wild-type polyester hydrolases PES-H1 and LCC and the engineered variant LCC-ICCG. Not only was TPA reliably detected by reproducible fold-increase in bioluminescence values in independently carried out assay set-ups under HT conditions (Figures 2C and 3); samples containing terephthalate from PET hydrolysis by PES-H1, LCC, and LCC-ICCG exhibited steady fold-increases over 4 h under HT assay conditions in *E. coli* RARE, which was in agreement with HPLC data. This sufficed to distinguish between wild-type enzymes and a variant with increased PET degradation activity and offers a semiquantitative screening tool for PET hydrolase libraries in the future.

Lastly, with the biocatalytic production of TAL from TPA, we accessed a highly reactive aldehyde intermediate that could be transformed into the corresponding primary diamine, for example, in aqueous reaction media (Figure 4A). The chemo-enzymatic three-step cascade also yielded >30% BAM from benzoic acid via the aldehyde and aldoxime intermediates (Figure 4B).

In conclusion, the presented work featured a complementary biosensor tool for the HT detection of TPA in living cells and suggested new routes for the bio-based interconversion of polymer building blocks, supporting efforts toward a circular plastic economy, the reduction of CO_2_ emissions, and the stewardship of resources.

### Limitations of the study

Although the utilization of *E. coli* BL21(DE3) Δ*lpp* significantly increased the CAR-catalyzed conversion of TPA *in vivo*, it was not superior to the established *E. coli* RARE strain for the LuxAB-based detection of aldehydes over longer reaction times because of their different metabolic backgrounds. However, the reproducible detection of TPA by the CAR_*Mm*_/LuxAB-coupled biosensor under HT assay conditions in *E. coli* RARE enabled the semiquantitative assessment of terephthalate salts in the supernatants obtained from the biocatalytic degradation by various PET hydrolases. Even though calculated yields were in the same concentration range according to calibrated HPLC, discrepancies arise from operating the biosensor system in whole-cells of *E. coli* because of the cytotoxicity of TPA and the corresponding aldehydes, for example. Accordingly, the bioluminescence yielded by the LuxAB-catalyzed reaction is transient and influenced by the metabolic background, the viability and physiological state of cells including aeration; because LuxAB is a monooxygenase, the generation of bioluminescence depends on the aldehyde substrate and O_2_. In addition, expression levels of enzymes, intracellular cofactor availability, and the background luminescence in living cells can add to variations but are easily addressed by appropriate (negative) controls, and the normalization of bioluminescence signals as discussed in detail previously (Bayer et al., 2021).

The interconversion of TPA into 1,4-bis-AMB through a three-step chemo-enzymatic cascade operating in one-pot only yielded only 15.0 ± 5.0% of the diamine and could not be improved by employing *E. coli* RARE, for example. The poor recovery of material (<50%) can be explained by the low solubility of TPA in aqueous solutions and the volatility of reaction intermediates. Furthermore, the formation of imines from aldehyde and amine precursors in aqueous solutions has been reported (Godoy-Alcántar et al., 2005; Simion et al., 2001) and will be investigated as a contributing factor in the future. Nonetheless, the reductive amination could be achieved in an aqueous buffer system, which advances the original protocol (Ayedi et al., 2013) and puts it in the context of transforming PET-derived TPA.

## STAR★Methods

### Key resources table

**Table:** 

REAGENT or RESOURCE	SOURCE	IDENTIFIER
**Bacterial and virus strains**		

*E. coli* BL21(DE3)	Thermo Scientific™	Cat#EC0114
*E. coli* BL21(DE3) Δ*lpp*	This paper	N/A
*E. coli* DH5α	Thermo Scientific™	Cat#18265017
*E. coli* K-12 MG1655 RARE	Prof. K.L.J. Prather (Kunjapur et al., 2014)	Addgene Bacterial strain #61440

**Chemicals, peptides, and recombinant proteins**		

PET film	Goodfellow GmbH	Cat#ES301445
Terephthalic acid (TPA; CAS: 100-21-0)	Sigma-Aldrich	Cat#185361
4-Carboxybenzaldehyde (4-CBAL; CAS: 619-66-9)	Acros	Cat#154580050
4-(Hydroxymethyl) benzoic acid (4-HMBA; CAS: 3006-96-0)	Sigma-Aldrich	Cat#382639
Terephthalaldehyde (TAL; CAS: 623-27-8)	Alfa Aesar	Cat#A14930
4-(Hydroxymethyl) benzaldehyde (4-HMBAL; CAS: 52010-97-6)	Carbosynth Ltd	Cat#FH140138
1,4-Benzenedimethanol (1,4-BDM; CAS: 589-29-7)	TCI	Cat#D0605
1,4-bis-(Aminomethyl) benzene (1,4-bis-AMB; CAS: 539-48-0)	Sigma-Aldrich	Cat#8.41656
Benzoic acid (CAS: 65-85-0)	Sigma-Aldrich	Cat#242381
Benzaldehyde (CAS: 100-52-7)	Acros	Cat#378361000
Benzyl alcohol (CAS: 100-51-6)	Fluka	Cat#77013
Benzylamine (BAM; CAS: 100-46-9)	Sigma-Aldrich	Cat#185701
Methyl benzoate (CAS: 93-58-3)	Sigma-Aldrich	Cat#M29908
2-Phenylacetic acid (2-PAA; CAS: 103-82-2)	Fluka	Cat#78490
2-Phenylacetaldehyde (2-PAAL; CAS: 122-78-1)	Acros	Cat#37091
2-Phenylethanol (CAS: 60-12-8)	Fluka	Cat#77861
Lysonase™ Bioprocessing Reagent	Merck-Millipore	Cat#71230
ROTI®Garose-His/Co Beads	Carl Roth	Cat#1235.1
Recombinant protein (C-term. 6xHis, purified): leaf-branch compost cutinase (LCC)	This study	G9BY57
Recombinant protein (C-term. 6xHis, purified): leaf-branch compost cutinase variant (LCC-ICCG)	This study	PDB: 6THT
Recombinant protein (C-term. 6xHis, purified): polyester hydrolase-1 (PES-H1)	This study	PDB: 7CUV
Q5® polymerase	NEB	Cat#M0491S
Q5® mutagenesis kit	NEB	Cat#E0554S

**Deposited data**		

Raw and analyzed data	This paper	N/A

**Experimental models: Organisms/strains**		

*E. coli* strains, see above: Bacterial and virus strains	This paper	N/A

**Oligonucleotides**		

*lpp*-up_F, primer for strain engineering, see Table S1	This paper (Thermo Scientific™)	N/A
*lpp*-up_R, primer for strain engineering, see Table S1	This paper (Thermo Scientific™)	N/A
*lpp*-down_F, primer for strain engineering, see Table S1	This paper (Thermo Scientific™)	N/A
*lpp*-down_R, primer for strain engineering, see Table S1	This paper (Thermo Scientific™)	N/A
pTarget_F, primer for strain engineering, see Table S1	This paper (Thermo Scientific™)	N/A
pTarget_R, primer for strain engineering, see Table S1	This paper (Thermo Scientific™)	N/A
Δ*lpp*-gRNA_F, primer for strain engineering, see Table S1	This paper (Thermo Scientific™)	N/A
Δ*lpp*-gRNA_R, primer for strain engineering, see Table S1	This paper (Thermo Scientific™)	N/A

**Recombinant DNA**		

Plasmid: pCDFduo/*luxAB*	Bayer et al., 2021	NCBI: WP_088373098 (*luxA*); NCBI: P19840 (*luxB*)
Plasmid: pACYCDuet-1/*car*_*Mm*_*:ppt*_*Ni*_	Bayer et al., 2021	NCBI: WP_012393886 (*car*_*Mm*_); NCBI: ABI83656 (*ppt*_*Ni*_)
Plasmid: pET26b/*lcc*	This paper (BioCat GmbH); (Tournier et al., 2020)	NCBI: G9BY57
Plasmid: pET26b/*lcc-ICCG*	This paper (BioCat GmbH); (Tournier et al., 2020)	PDB: 6THT
Plasmid: pET26b/*pes-H1*	This paper (BioCat GmbH); (Zimmermann et al., 2019)	PDB: 7CUV
Plasmid: pCas	Addgene (Jiang et al., 2015)	Addgene Plasmid #62225
Plasmid: pTarget	Addgene (Jiang et al., 2015)	Addgene Plasmid #62226
Plasmid: pTarget-Δ*lpp*	This paper	N/A

**Software and algorithms**		

Geneious Prime® 2022.0.2	Biomatters Ltd	www.geneious.com
OligoEvaluator™	Sigma-Aldrich	http://www.oligoevaluator.com/LoginServlet
Microsoft Office 16.0	Microsoft Corporation	www.microsoft.com

**Other**		

96-well plate (flat bottom, black polystyrene)	Greiner Bio-One	Cat#655079

### Resource availability

#### Lead contact

Further information and requests for resources and reagents should be directed to and will be fulfilled by the lead contact, Dr. Thomas Bayer (thomas.bayer@uni-greifswald.de).

#### Materials availability

• For the assembly of the pTarget-Δ*lpp* plasmid in this study, the templates pCas (#62225) and pTarget (#62226) were purchased from Addgene (Watertown, USA). Subsequently, pTarget-Δ*lpp* and pCas were used to knock-out the *lpp* gene from the genome of *E. coli* BL21(DE3). The genes encoding the leaf-branch compost cutinase (LCC) and the LCC-ICCG variant (Tournier et al., 2020) and the polyester hydrolase-1 (PES-H1) (Zimmermann et al., 2019) were codon-optimized for the expression in *E. coli*, synthesized, and cloned in frame with the C-terminal 6xHis tag present in pET26b by the BioCat GmbH (Heidelberg, Germany). Accession numbers of proteins are provided in the Key resources table.
• *E. coli* BL21(DE3) (#EC0114) and DH5α (#18265017) were initially purchased from Thermo Scientific™ (Darmstadt, Germany) and propagated as described below. *E. coli* BL21(DE3) Δ*lpp* is available from the lead contact upon request. *E. coli* RARE was acquired from the Prather group (Kunjapur et al., 2014) but is also available from Addgene (#61440).
• There are restrictions to the availability of the previously constructed pCDFduo/*luxAB*, herein referred to as pLuxAB, and pACYCDuet-1/*car*_*Mm*_*:ppt*_*Ni*_ plasmids (Bayer et al., 2021) due to material transfer agreements (MTAs). Further information is available from the lead contact upon request.
• Otherwise, this study did not generate new unique reagents.

### Experimental model and subject details

*E. coli* BL21(DE3), *E. coli* BL21(DE3) Δ*lpp*, *E. coli* DH5α, and *E. coli* RARE were propagated in 4–5 mL lysogeny broth (LB) medium (25 g L^−1^; Sigma-Aldrich, Buchs, Switzerland) in Infors HT Multitron incubator shakers (Bottmingen, Switzerland) at 37°C with shaking (150–180 rpm) for 12–16 h. If not stated otherwise, chemically competent *E. coli* cells were produced by using 0.1 M CaCl_2_ and transformed with plasmid DNA (25–100 ng) by heat-shock at 42°C for 45 s as previously described (Bayer et al., 2021). For the efficient transformation of *E. coli* RARE, plasmids were passed through *E. coli* DH5α (Bayer et al., 2021). *E. coli* transformants harboring pLuxAB and pACYCDuet-1/*car*_*Mm*_*:ppt*_*Ni*_ were propagated in LB medium supplemented with streptomycin (25 μg·mL^-1^) and chloramphenicol (34 μg·mL^-1^), respectively. Only half the concentration of antibiotics was used for the selection and subsequent propagation of strains harboring both plasmids. For the selection and propagation on plates, LB containing 1.5% (*ω/ν*) agar (Carl Roth, Karlsruhe, Germany) and supplemented with antibiotics – if applicable – was used.

### Method details

#### Strain engineering

The non-essential *lpp* gene encodes a cellular ‘*bulk*’ protein (Li et al., 2014), which controls the (mechanical) properties of the inner and outer membrane and the width of the periplasmic space (Asmar et al., 2017; Mathelié-Guinlet et al., 2020). The deletion of the *lpp* gene from the *E. coli* genome has been suggested to affect the permeability of the cellular envelope for small molecules (Ni et al., 2007) that might also influence the uptake of TPA and derivatives. Furthermore, expression levels of CAR_*Mm*_/PPT_*Ni*_ were increased in *E. coli* BL21(DE3) Δ*lpp* according to SDS-PAGE analysis (Figure S2). This may be due to the re-allocation of cellular resources (Li et al., 2014).

*E. coli* BL21(DE3) Δ*lpp* was constructed by using a previously developed two-plasmid-based CRISPR/Cas9 system (Jiang et al., 2015). The two key plasmids, pCas (#62225) and pTarget (#62226), were purchased from Addgene (Watertown, USA). The pTarget-Δ*lpp* plasmid was constructed by first engineering the flanking sequence of the *lpp* gene by the assembly of three DNA fragments using a sequence- and ligation-independent cloning extract-based protocol (Zhang et al., 2012): (1) the *lpp*-up fragment (amplified from the *E. coli* genome employing the primers *lpp*-up_F/R), (2) the *lpp*-down fragment (amplified from the genome of *E. coli* using the primers *lpp*-down_F/R), and (3) the pTarget fragment. The latter was amplified by using the primers pTarget_F/R. Next, the guide RNA (gRNA) was introduced employing the Q5® mutagenesis kit (New England Biolabs, Frankfurt/Main, Germany) with the primer pair Δ*lpp*-gRNA_F/R. The resulting pTarget-Δ*lpp* was Sanger sequenced (Eurofins Genomics, Ebersberg, Germany) to confirm the insertion of gRNA and the flanking sequences of the *lpp* gene. Subsequently, *E. coli* BL21(DE3) Δ*lpp* strain was constructed by using pCas and pTarget-Δ*lpp* according to Jiang and co-workers (Jiang et al., 2015). Briefly, competent *E. coli* BL21(DE3) cells were transformed with pCas as described above. In the resulting pCas transformants, the λ-Red system was induced with 0.2% (*ω/ν*) arabinose and electrocompetent cells were prepared. Next, the pTarget-Δ*lpp* plasmid was introduced by electroporation; transformants were selected on LB agar plates supplemented with kanamycin and streptomycin. Colony polymerase chain reaction (PCR) was performed for genotyping the colonies. The plasmids pTarget-Δ*lpp* and pCas were cured sequentially in the presence of 0.5 mM isopropyl-β-D-thiogalactopyranoside (IPTG) and by culturing at 37°C, respectively. Finally, the successful construction of *E. coli* BL21(DE3) Δ*lpp* was confirmed by PCR amplification and DNA sequencing of the genome sequence flanking the knocked-out *lpp* gene.

Desalted DNA oligonucleotides were ordered from Invitrogen/Thermo Fisher Scientific and dissolved in nuclease-free water (Invitrogen, Darmstadt, Germany). Primer sequences are given in Table S1. PCRs were performed on a Biometra TAdvanced thermal cycler (Analytik Jena, Jena, Germany) employing Q5® polymerase as suggested by the supplier (New England Biolabs).

#### Enzyme production and resting cell preparation

Production of LuxAB and CAR_*Mm*_/PPT_*Ni*_ was performed in *E. coli* transformants harboring pLuxAB and pACYCDuet-1/*car*_*Mm*_*:ppt*_*Ni*_, respectively. For cultivation, auto-induction medium (AIM; 2.5% (*ω/ν*) lysogeny broth medium, 1 mM MgSO_4_, 25 mM (NH_4_)_2_SO_4_, 50 mM KH_2_PO_4_, 50 mM Na_2_HPO_4_, 5% (*ω/ν*) glycerol, 0.5% (*ω/ν*) glucose, 2% (*ω/ν*) α-lactose) supplemented with chloramphenicol (34 μg·mL^-1^) and streptomycin (25 μg·mL^-1^), respectively, was used. Only half the concentration of antibiotics was used for the selection and subsequent cultivation of strains harboring both plasmids. The AIM was adapted from Studier (Studier, 2005).

Briefly, a single colony of the desired strain was grown in LB medium supplemented with the appropriate antibiotic(s) at 37°C (180 rpm) for 12–16 h. AIM containing antibiotic(s) was inoculated with 0.2% (*ν/ν*) preculture in baffled flasks and incubated in Infors HT Multitron incubator shakers (Bottmingen, Switzerland) at 37°C (180 rpm) for 4–6 h (6 h for co-transformants, 5 h for cells harboring pLuxAB, and 4 h for cells harboring pACYCDuet-1/*car*_*Mm*_*:ppt*_*Ni*_). Enzyme production was performed at 20°C (150 rpm) for 16–20 h. The optical density at 600 nm (OD_600_) of cultures was determined with a UV-1280 spectrophotometer (Shimadzu, Kyoto, Japan). Cells were harvested by centrifugation (4,000 ✕ g, 4°C) for 15–20 min using a Heraeus Fresco 17 centrifuge or a Heraeus Labofuge 400R (Thermo Fisher Scientific) (Bayer et al., 2021).

RCs of *E. coli* were prepared after cultivation by re-suspension in RCM (22 mM KH_2_PO_4_, 42 mM Na_2_HPO_4_, 8.56 mM NaCl, 1 mM MgSO_4_, 1 mM CaCl_2_ and 1% (*ω/ν*) glucose) to a final OD_600_ ≈ 10.0 as previously described (Bayer et al., 2021).

Similarly, LCC, LCC-ICCG, and PES-H1 were produced in *E. coli* BL21(DE3) transformants by cultivation in AIM supplemented with kanamycin (50 μg·mL^-1^) at 21°C (150 rpm) for 23 h (Tournier et al., 2020). To produce PES-H1, the time of cultivation was reduced from 23 h to 20 h. Cells were harvested, re-suspended in lysis buffer (7 mL per g wet cell pellet; 50 mM Na_2_HPO_4_, 300 mM NaCl, pH 8), and processed according to Tournier and co-workers (Tournier et al., 2020). After cell disruption by freezing/thawing, 1 μL Lysonase^TM^ Bioprocessing Reagent (#71230; Merck-Millipore, Darmstadt, Germany) per mL cell suspension was added and incubated at 28°C (220 rpm) for 1 h. They lysate was clarified by centrifugation (6,000 ✕ g, 4°C) for 45 min. Subsequently, enzymes were purified from the supernatant through their C-terminal 6xHis tags by cobalt affinity chromatography (ROTI® Garose-His/Co Beads, Carl Roth, Karlsruhe, Germany). The three hydrolases were eluted with elution buffer (50 mM Na_2_HPO_4_, 300 mM NaCl, pH 8.0) supplemented with 250 mM (LCC and LCC-ICCG) or 100 mM imidazole (PES-H1). The target proteins were desalted with 50 mM Na_2_HPO_4_ buffer (pH 8.0) and used for the hydrolysis of Gf-PET film (Goodfellow, Hamburg, Germany) as describe below.

Protein expression was confirmed by 12.5% (*ω/ν*) SDS-PAGE of denatured whole-cell samples normalized to OD_600_ = 7.0 (Figure S2) or purified enzymes, using the Mini-PROTEAN electrophoresis system (Bio-Rad, Feldkirchen, Germany) and following standard protocols; gels were stained with InstantBlue^TM^ Protein Stain (Expedeon, Heidelberg, Germany) for at least 30 min (Bayer et al., 2021).

#### PET hydrolysis

The enzymatic degradation of amorphous PET film (Goodfellow, Hamburg, Germany) was performed in 1 M potassium phosphate buffer (pH 8.0) at 72°C with shaking (1 000 rpm; ThermoMixer C, Eppendorf, Hamburg, Germany) for 24 h; the enzyme concentration of 1 mg per g PET in a total reaction volume of 1.5 mL was used as published before (Tournier et al., 2020). The clarified supernatants were analyzed according to Palm et al. by reverse-phase HPLC on a VWR Hitachi LaChrom Elite system (VWR International, Radnor, USA), equipped with a Kinetex® column (5 μM EVO C18 100 Å, 150 x 4.6 mm; Phenomenex®, Aschaffenburg, Germany), with a gradient of acetonitrile and 0.1% (*ν/ν*) formic acid in water at 30°C after injection of 10 μL sample. Within 12 min, acetonitrile was increased from 5 to 44% and then to 70% after 15 min. The ratio remained constant for another 3 min. TPA was detected at 240 nm and quantification was facilitated by standard calibration using commercial reference compounds (Palm et al., 2019). Samples were prepared from independent PET hydrolysis experiments with LCC, LCC-ICCG, and PES-H1 and subsequent HPLC measurments and quantification (n ≥ 2; Table S2).

#### LuxAB-based detection of TPA-derived aldehydes *in vivo* (96-well plate format)

RCs of the desired *E. coli* strain expressing either LuxAB or LuxAB together with CAR_*Mm*_/PPT_*Ni*_ were prepared as before in biological replicates (n ≥ 3). To 198 μL RCs (OD_600_ ≈ 10.0) per well, 2 μL of the target compound, dissolved in DMSO, were added to a final concentration of 1 mM concentration, if not stated otherwise, in a total volume of 200 μL containing 1% (*ν/ν*) DMSO as co-solvent per well (flat bottom, black polystyrene 96-well plate, #655079; Greiner Bio-One, Frickenhausen, Germany). It was mixed gently. The bioluminescence was measured immediately on a Varioskan^TM^ LUX multimode plate reader (Thermo Fisher Scientific). The change in bioluminescence was monitored at 25°C for up to 1 h and the fold-increase in bioluminescence above background in the presence of directly added or enzymatically produced aldehydes calculated as described in detail previously (Bayer et al., 2021). Data were generated from biological replicates and presented as mean values + SD (n ≥ 3). These results are shown in Figures 2B–2C and Figure S3 for RCs of *E. coli* BL21(DE3) Δ*lpp* and *E. coli* RARE, respectively.

For the screening of PET hydrolysates, samples were clarified by centrifugation (13,000 ✕ g, 1 min) using a Heraeus Labofuge 400R (Thermo Fisher Scientific). The supernatant was diluted 1:10 by mixing 100 μL of the hydrolysis sample with 200 μL DMSO and 700 μL RCM. Subsequently, 10 μL of the resulting dilution were added to 190 μL RCs (OD_600_ ≈ 10.0) per well. The 96-well plate was processed as before and the bioluminescence was measured up to 4 h. Data were generated from biological replicates and presented as mean values + SD (n ≥ 3). These results are shown in Figures 3 and S4 for RCs of *E. coli* RARE and *E. coli* BL21(DE3) Δ*lpp*, respectively.

#### Whole-cell biotransformations and chemo-enzymatic cascade one-pot reaction

RCs (OD_600_ ≈ 10.0) expressing CAR_*Mm*_/PPT_*Ni*_ were prepared as before. Whole-cell biotransformations were performed in glass vials with screwcaps (4 mL) at 2–5 mM TPA concentration and 5% (*ν/ν*) DMSO as co-solvent (V_total_ = 0.5 mL) in Infors HT Multitron incubator shakers (Bottmingen, Switzerland) at 25°C (230–250 rpm) for 0–24 h. For GC analysis, samples (100 μL) of the biotransformation mixtures were taken immediately after the addition of substrate and mixing (t ≈ 0 h) and again after 24 h. Subsequently, samples were acidified with 2 M HCl (10 μL) and extracted two times with ethyl acetate (200 μL) containing 1 mM methyl benzoate as internal standard (IS) by vortexing for 1 min. For phase separation, samples were centrifuged (13,000 ✕ g, 4°C) for 1 min. The combined organic phases were desiccated over Na_2_SO_4_ and transferred into a GC vial with insert, capped, and submitted to GC analysis. Compound identification was performed by the comparisons of retention times of commercial standards (Table 1), unless stated otherwise; quantification and calculation of GC yields were performed by employing relative response factors (RRFs) as described in detail previously (Bayer et al., 2021) and below. Data were generated from biological replicates and presented as mean values + SD (n ≥ 3). When employing RCs of *E. coli* BL21(DE3) or *E. coli* RARE, reaction mixtures contained mainly unreacted TPA after 24 h (58.2 ± 16.0% for *E. coli* BL21(DE3) and 51.0 ± 12.1% for *E. coli* RARE as shown in Figures S1A and S1B, respectively). Conversions of TPA could be improved by the utilization of *E. coli* BL21(DE3) Δ*lpp*. After 24 h reaction time, biotransformation mixtures only contained 31.1 ± 5.9% TPA, besides TPA-derived aldehydes including 4-CBAL and the over-reduced 4-HMBAL and 1,4-BDM (Figure 2A). Additionally, 4-CBAL and 4-(hydroxymethyl) benzoic acid (4-HMBA) were identified as substrates for CAR_*Mm*_ as shown in Figures S1C and S1D, respectively. These results are in agreement with the LuxAB-based detection of corresponding aldehydes employing either RCs of *E. coli* BL21(DE3) Δ*lpp* (Figure 2) or *E. coli* RARE (Figure S4) in the HT assay. Furthermore, whole-cell biotransformations suggest the activity of endogenous enzymes (e.g., aldehyde dehydrogenases) that oxidize aldehydes to the corresponding carboxylic acids (Figures S1C–S1E). This is in accordance with previous findings (Bayer et al., 2017).

For the chemo-enzymatic reaction in one-pot, TPA was reduced by CAR_*Mm*_ in whole-cell biotransformations under the conditions given above (V_total_ = 0.1 mL) in the presence of 2.2–2.5 eq NH_2_OH · HCl in biological replicates (n ≥ 2). After 12–16 h of reaction time, 5.5 eq zinc powder were added and the cell suspension acidified with 10 M HCl (20 μL). After incubation with shaking at room temperature for 4 h, 30% (ν/ν) ammonia solution (10 μL) and 5 M NaOH (10 μL) were added (Ayedi et al., 2013). It was mixed for 15 min before extracting two times with ethyl acetate (200 μL) containing 1 mM IS as before. Combined organic phases were dried over Na_2_SO_4_ and submitted to GC analysis using a GC-2010 Plus (Shimadzu) equipped with a flame ionization detector (FID; Shimadzu) and a ZB-5MSi column (length: 30 m; inner diameter: 0.25 mm; film thickness: 0.25 μm) from Phenomenex (Torrance, USA). GC/FID method (hydrogen, 0.96 mL·min^-1^ flow rate; injector and detector: 320°C): 100°C, hold 1 min, 20°C per min to 250°C, hold 5 min; total time: 13.5 min.

### Quantification and statistical analysis

Quantification was performed for (1) biocatalytic reactions performed in whole cells of *E. coli* (i.e., RCs) in biological replicates (n ≥ 3) and in combination with reductive amination reactions in one-pot (n ≥ 2) and (2) the enzymatic degradation of PET for independent hydrolysis reactions (n ≥ 2). Compound identification was realized by the comparison of the retention times of commercial standards by (1) GC/FID and (2) HPLC analysis. From the corresponding peak areas, yields were calculated (1) by employing RFFs for each compound of interest (Table 1) and (2) by linear standard calibration for TPA (slope = 49,134.00; axis intercept = 58,547.00; R^2^ > 0.99).

For the semi-quantitative assessment of amounts of TPA in PET hydrolysis samples, dilutions were analyzed by the CAR/LuxAB-based HT assay employing biological replicates of RCs (n ≥ 3). The fold-increase in bioluminescence was proportional to the concentration of TPA as described in the main text and could be calculated from TPA samples with known concentration (1 mM).

Statistical analysis included the calculations of mean values, SDs, and the determination coefficient (R^2^) by the integrated functions of the standard spreadsheet software Microsoft Excel (version 16.0).

GC yields are presented as bars representing mean values + SD in Figures 2A, 4, and S1. Both experimental and statistical details can also be found in the corresponding figure legends and in the main text.

HPLC yields are given as mean values ± SD in Table S2. Experimental details can be found in the main text and statistical details in the legend of Table S2.

The mean fold-increase in bioluminescence + SD is depicted as bars in Figures 3 and S4. Both experimental and statistical details can also be found in the corresponding figure legends and in the main text.

### Additional resources

The OligoEvaluator^TM^ (Sigma-Aldrich; http://www.oligoevaluator.com/LoginServlet) was used to predict secondary structures and dimer formation of DNA oligonucleotides (Table S1).

## Data Availability

• The genome of *E. coli* BL21(DE3) and associated metadata were retrieved from the National Center for Biotechnology Information (NCBI; GenBank: CP001509.3). The accession numbers of protein sequences are listed in the Key resources table. • This paper does not report original code. • Any additional information required to re-analyze the data reported in this paper is available from the lead contact upon request.
