# Supplementary material for: Biosensor and chemo-enzymatic one-pot cascade applications to detect and transform PET-derived terephthalic acid in living cells
Source: iScience. 2022 Apr 29;25(5):104326. doi: 10.1016/j.isci.2022.104326 (PMC9117539; doi:10.1016/j.isci.2022.104326)
Supplement: Document S1. Figures S1–S4 and Table S1 and S2 [file mmc1.pdf]

iScience, Volume *25*

Supplemental information

Biosensor and chemo-enzymatic one-pot cascade applications to detect and transform PET-derived terephthalic acid in living cells

Thomas Bayer, Lara Pfaff, Yannick Branson, Aileen Becker, Shuke Wu, Uwe T. Bornscheuer, and Ren Wei

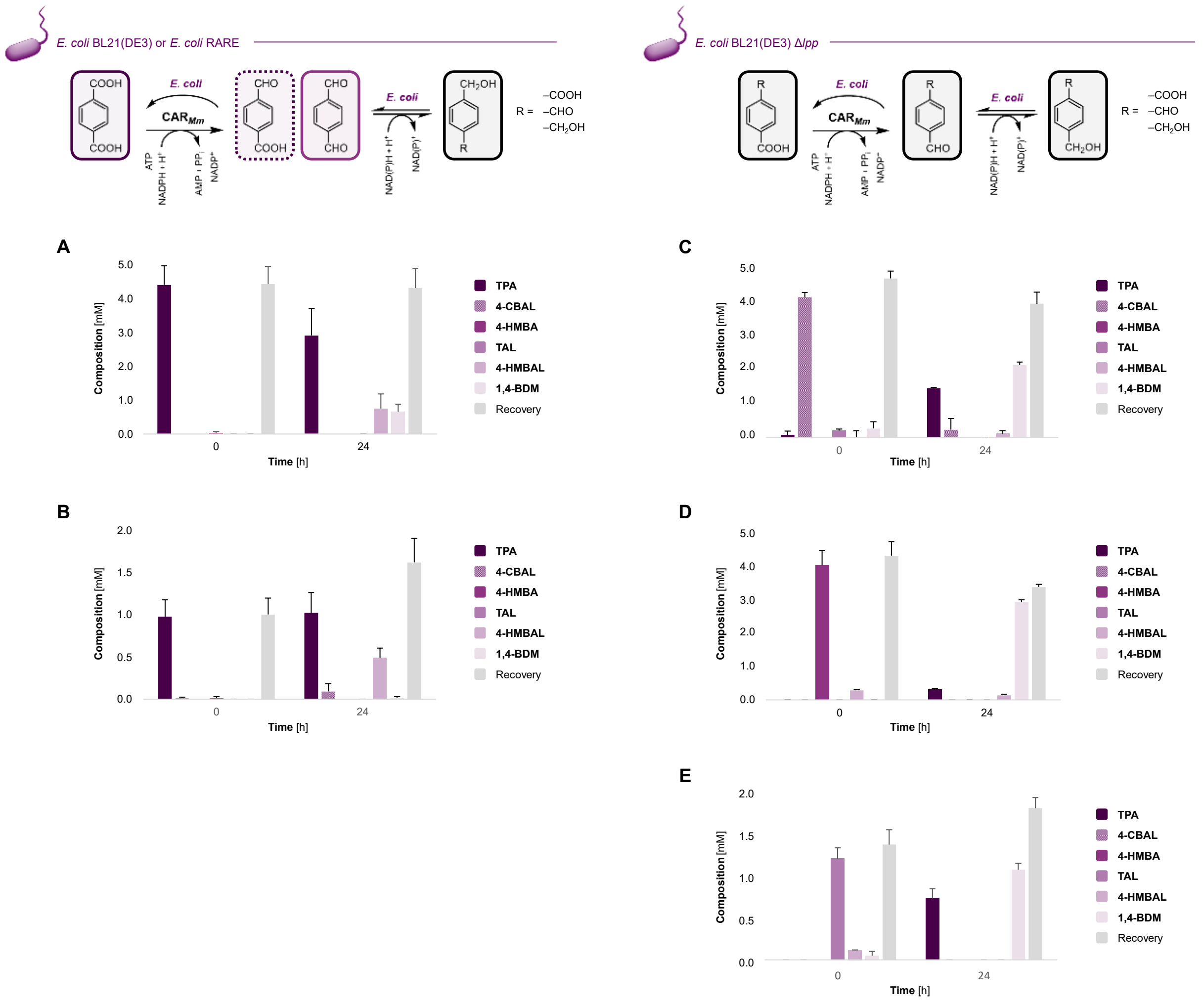

Figure S1. CAR-catalyzed reductions of TPA and derivatives *in vivo*, related to Figure 2A. (**A**) Bioreduction of TPA by CAR$_{Mm}$ in *E. coli* BL21(DE3) yields a mixture of the over-reduced 4-HMBAL and 1,4-BDM, besides unreacted substrate. (**B**) Bioreduction of TPA by CAR$_{Mm}$ in *E. coli* RARE mainly yields 4-HMBAL, indicating reduced aromatic aldehyde reducing activity compared to *E. coli* BL21(DE3), 4-CBAL, and unreacted TPA. (**C**) CAR$_{Mm}$ reduces the carboxylate group in 4-CBAL to the corresponding TAL. Endogenous enzymes not only reduce aldehydes to the corresponding primary alcohols like 1,4-BDM but also oxidize them to the carboxylates as indicated by the detection of TPA after 24 h. This is in accordance with previous findings (Bayer et al., 2017). (**D**) CAR$_{Mm}$ reduces the carboxylate group in 4-HMBA to the corresponding 4-HMBAL. Endogenous enzymes reduce the aldehydes to the corresponding primary alcohols like 1,4-BDM; TPA could be detected in traces after 24 h. (**E**) The highly reactive TAL is both oxidized and reduced by endogenous enzymes, yielding TPA and 1,4-BDM, respectively. On top, arrows indicate the activities of (host) enzymes; PPT$_{Ni}$ necessary for posttranslational modification of CAR$_{Mm}$ is omitted for clarity. Experiments were performed in RCs of *E. coli* (OD_{600} ≈ 10.0) co-expressing enzymes from pACYCDuet-1/*car*$_{Mm}$:*ppt*$_{Ni}$ (Bayer et al., 2021) in the presence of 2–5 mM substrates and 5% (*v/v*) DMSO as organic co-solvent. Sampling: 0 h (after the addition of substrate and mixing) and 24 h. Recoveries were reduced due to low solubilities and/or the volatility of compounds. GC yields are presented as mean values + standard deviation (SD) [mM] of biological replicates (n ≥ 3).

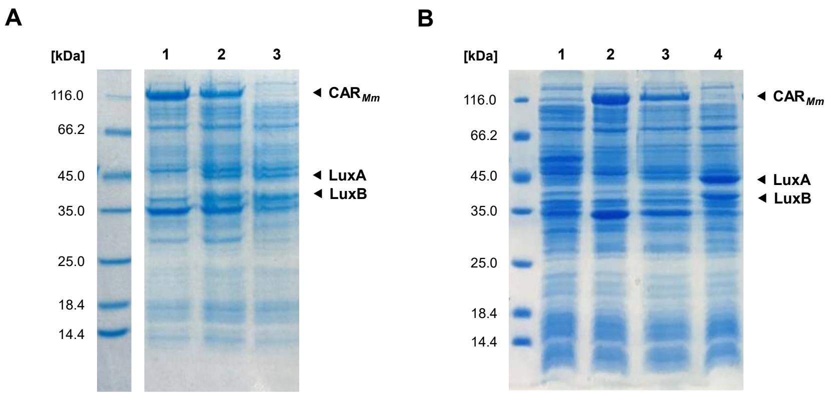

Figure S2. SDS-PAGE analysis of whole-cell samples, related to Figure 2A, Figure S1, and STAR Methods. (**A**) Expression of (**1**) CAR$_{Mm}$/PPT$_{Ni}$ from pACYCDuet-1/*car*$_{Mm}$:*ppt*$_{Ni}$ [CAR$_{Mm}$: 129 kDa], (**2**) CAR$_{Mm}$/PPT$_{Ni}$ and LuxAB from pLuxAB [LuxA: 43 kDa, LuxB: 37 kDa], and (**3**) LuxAB in *E. coli* BL21(DE3). (**B**) Whole-cell samples of (1) untransformed *E. coli* BL21(DE3) Δlpp or expressing (**2**) CAR$_{Mm}$/PPT$_{Ni}$, (**3**) CAR$_{Mm}$/PPT$_{Ni}$ and LuxAB, and (**4**) LuxAB; PPT$_{Ni}$ [23 kDa] was not detectable due to low expression levels in corresponding samples. Proteins were produced from pACYCDuet-1/*car*$_{Mm}$:*ppt*$_{Ni}$ and pLuxAB (Bayer et al., 2021); the detailed protocol is given in the main text. Sample loading normalized to OD_{600} = 7.0; SDS-PAGE and gel staining performed as described in the main text. Irrelevant lanes were cropped in (**A**), the brightness of both pictures was increased by 20%; (◂) indicate protein bands of interest.

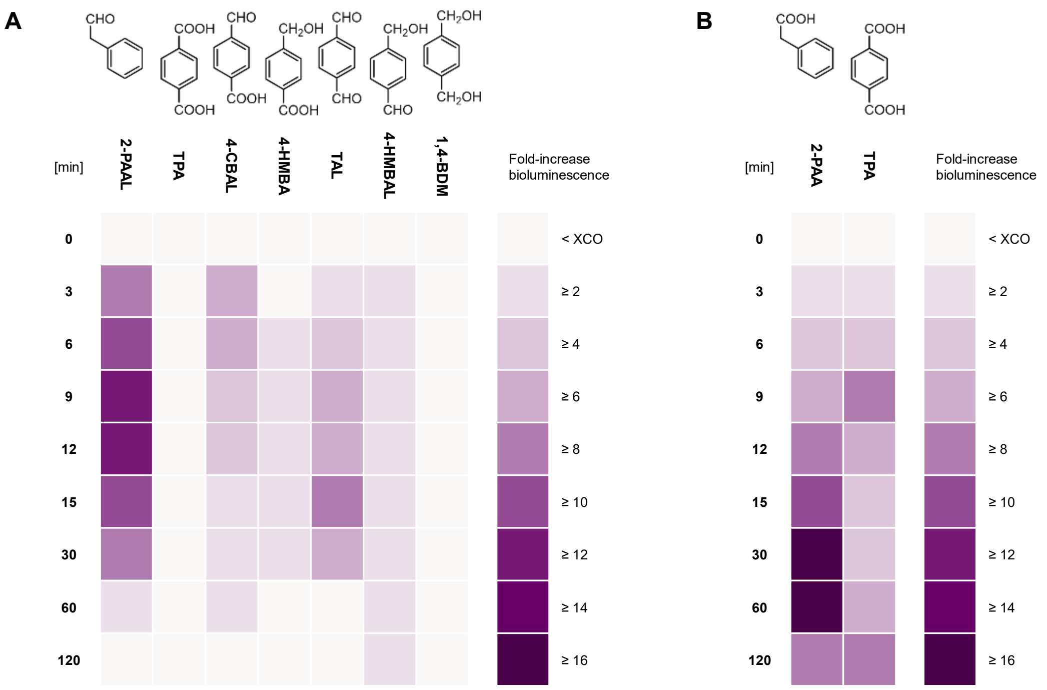

Figure S3. LuxAB-based HT detection of aldehydes in *E. coli* RARE, related to Figure 2B–C. (**A**) Direct detection of TPA-derived aldehydes (0.1 mM) by increasing bioluminescence over time in RCs of *E. coli* RARE expressing LuxAB from pLuxAB; 2-PAAL was used as the positive control. Whereas TPA and 1,4-BDM did not yield bioluminescence, the addition of 4-HMBA yielded background luminescence at 1 mM final concentration. (**B**) *In situ* production of aldehydes from 2-PAA and TPA (1 mM) in RCs of *E. coli* RARE co-expressing LuxAB and CAR_{Mm}/PPT_{Ni}. Experiments were performed in the presence of 1% (*v/v*) DMSO under HT assay conditions as described previously (Bayer et al., 2021) and in the main text; data presented as mean fold-increase bioluminescence obtained from biological replicates (n = 3).

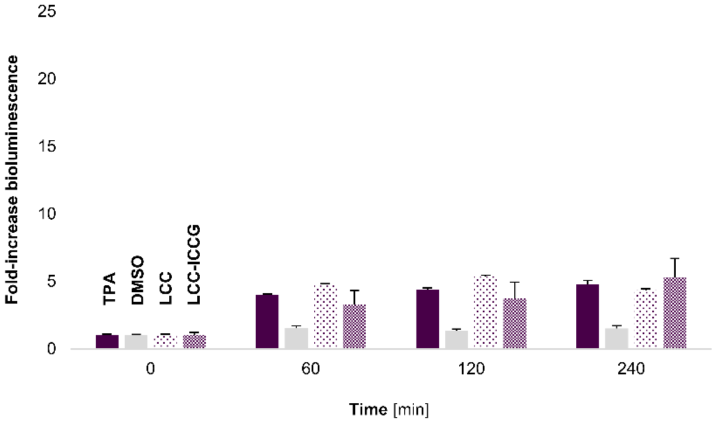

Figure S4. PET hydrolysis samples analyzed under HT conditions in *E. coli* BL21(DE3) Δ*lpp*, related to Figure 3. The enzyme-coupled biosensor system yielded bioluminescence in the presence of 1 mM TPA (positive control) and hydrolysates obtained by the enzymatic degradation of Gf-PET films by wildtype LCC and the LCC-ICCG variant. The bioluminescence did not increase in the presence of 1% (v/v) DMSO over monitoring time (negative control). While the bioluminescence plateaued around 4-fold above background in *E. coli* BL21(DE3) Δ*lpp* after 1 h incubation time, it increased in RCs of *E. coli* RARE proportionally to the amounts of TPA present in PET hydrolysis samples (see **Figure 3** in the main text). Experiments were performed in RCs of *E. coli* BL21(DE3) Δ*lpp* under HT assay conditions as described previously (Bayer et al., 2021) and in the main text; data presented as mean values of the fold-increase in bioluminescence + SD of biological replicates (n = 3).

Table S1. List of DNA oligonucleotides, related to **STAR Methods**

Primer	Sequence (5' – 3')
lpp-up_F	gagtcgacctgcagaagcttGTAAAGAACTGGCTCTGCAGAG
lpp-up_R	acaggtactaCCCTCTAGATTGAGTTAATCTCC
lpp-down_F	atctagagggTAGTACCTGTGAAGTGAAAAATG
lpp-down_R	gagctgcacatgaactcgagATGAATGCACCGGATATTAAAGC
pTarget_F	ctcgagttcatgtgcagctc
pTarget_R	aagcttctgcaggtcgactc
Δ*lpp*-gRNA_F	AGTAGAACCCgttttagagctagaaatagcaagtt
Δ*lpp*-gRNA_R	CTGCTGGCAGactagtattatacctaggactgagc

Table S2. HPLC yields of TPA in PET hydrolysates, related to **Figure 3** and **STAR Methods**

PET hydrolase	TPA yields [mM]
PES-H1	56.0 ± 0.1
LCC	47.8 ± 3.1
LCC-ICCG	111.1 ± 15.3

Yields are given as mean values ± SDs of independent PET hydrolysis experiments and subsequent HPLC measurement for LCC (n = 3) and LCC-ICCG (n = 2) and as mean value ± SD of a technical replicate (n = 2) for PES-H1.
